# Macro and trace elements signature of periodontitis in saliva: A systematic review with quality assessment of ionomics studies

**DOI:** 10.1111/jre.12956

**Published:** 2021-11-27

**Authors:** Giacomo Baima, Giovanni Iaderosa, Matteo Corana, Federica Romano, Filippo Citterio, Agnese Giacomino, Giovanni N. Berta, Mario Aimetti

**Affiliations:** ^1^ Department of Surgical Sciences C.I.R. Dental School University of Turin Turin Italy; ^2^ Department of Drug Science and Technology University of Turin Turin Italy; ^3^ Department of Clinical and Biological Sciences University of Turin Turin Italy

**Keywords:** biomarkers, diagnosis, essential nutrients, ionomics, periodontal disease

## Abstract

**Objectives:**

The present systematic review examined the available evidence on distinctive salivary ion profile in periodontitis compared to periodontal health and provided a qualitative assessment of the literature.

**Background:**

Macro and trace elements are essential for cellular physiology, and their changes in biological fluids can be revelatory of an underlying pathological status.

**Methods:**

Data from relevant studies identified from PubMed, Embase, and Scopus databases were retrieved to answer the following PECO question: *“In systemically healthy individuals, are there any differences in any salivary macro or trace element concentration between periodontally healthy subjects (H) and patients with periodontitis (P)?”* Quality of included studies was rated using a modified version of the QUADOMICS tool. A consistency analysis was performed to identify significantly discriminant chemical elements.

**Results:**

After the screening of 873 titles, 13 studies were included reporting data on 22 different elements. Among them, levels of sodium and potassium were consistently and significantly higher in P compared to H. Conflicting results were found for all the other elements, despite concentration of calcium, copper, and manganese mostly increased in saliva of P. Levels of magnesium were found higher in P than in H in 2 studies but lower in 3. Zinc resulted significantly increased in saliva from H compared to P individuals in 2 studies, but one study reported opposite results. Four studies were considered as high quality, while reporting of operative protocols and statistical analysis was a major limitation for the others. Due to high methodologic heterogeneity, meta‐analysis was not performed.

**Conclusions:**

Levels of macro or trace elements were differentially identified in saliva across diverse periodontal conditions, having a major potential for investigation of oral homeostasis and for high‐resolution periodontal diagnosis. Products of inflammatory physiologic cellular impairment, such as sodium and potassium, were the most consistently associated with periodontitis (PROSPERO CRD42021235744).

## 1 INTRODUCTION

Periodontitis is one of the most prevalent chronic non‐communicable diseases worldwide,^1^ affecting up to 20% of the people over 40 years of age in its severe form.^2^ When periodontitis occurs, the tissues that surround and support the teeth undergo an irreversible destruction that may progress over time, eventually leading to tooth migration or tooth loss with consequent disability in terms of altered masticatory function and aesthetics, and impairment of general health.^3^, ^4^ Early detection of periodontitis would be the key to preventing a more severe illness for the patient and to reducing the economic burden for the society. Indeed, treatment of periodontitis at its initial stages is easy and successful, whereas at more advanced stages, it requires surgical procedures performed by trained specialists to arrest disease progression. Therefore, non‐invasive accurate diagnostic methods are sought to discriminate periodontitis from healthy subjects in a rapid way which may be accessible to large proportions of the population.^5^

Salivary diagnostics is evolving as a highly potential field for early diagnosis and monitoring of oral and systemic diseases.^6^, ^7^ Saliva can be obtained by inexpensive sampling methods that cause minimal patient discomfort and contains a vast variety of biomolecules, such as DNA, mRNA, microRNA, protein, metabolites, microbiota, and ions.^8^ Thus, it represents an ideal fluid for the discovery and validation of biomarkers.^9^ Although many clinical applications of salivary diagnostics are available to date, the lack of definite biomarkers for periodontal disease and portable, inexpensive, user‐friendly diagnostic platforms still prevent salivary diagnostics from being fully clinically translated.

Ionomics is the study of the ionome, defined as “the mineral nutrient and trace element composition of an organism, representing the inorganic component of cellular and organismal systems.”^10^ Some macro elements are essential for the human life, and they are needed in more amount because they control important biological processes, such as hormone synthesis, cellular signaling, and enzymatic catalysis (such as magnesium, calcium, potassium, and sodium). Some other elements (such as zinc, iron, selenium, and iodine) are required in very small amounts and, therefore, are known as trace elements.^11^ In the last years, ionomics has been increasingly applied to the investigation of human physiologic and pathological conditions.^12^, ^13^ Imbalance in essential elements has been consistently associated with human diseases, such as diabetes mellitus, neurodegenerative diseases, cancer, and periodontitis.^14^

Recently, research interest has been directed toward the analysis of the salivary mineral profile across different periodontal conditions considering that macro and trace mineral deficiency or excess could be related to both inflammation and oxidative damage, and they may be associated with periodontal tissue breakdown. Since no systematic reviews have been conducted to date, the present study aimed at summarizing the available evidence regarding the feasibility of salivary ionomic analysis for the diagnosis of periodontitis. Moreover, a methodologic quality assessment of the included studies was provided in order to evaluate risk of bias and favor a standardization of future research endeavors.

## 2 MATERIALS AND METHODS

This research has been conducted according to the Cochrane Handbook and reported according to the PRISMA statement.^15^, ^16^ The protocol was registered on PROSPERO (CRD42021235744).

### 2.1 Focused questions

This systematic review was designed to answer the following focused PECO question:

In systemically healthy individuals, are there any differences in any salivary macro or trace element concentration between periodontally healthy subjects and patients with periodontitis?

(P) Population. Adult patients in good systemic health.

(E) Exposure. Patients with a clinical diagnosis of periodontitis (P).

(C) Comparison. Subjects with periodontal health (H) or gingivitis (G).

(O) Type of outcome measures. Difference in salivary macro or trace element levels between P patients and G or H controls.

### 2.2 Eligibility criteria

#### 2.2.1 Types of studies

Original studies in humans with observational design (cross‐sectional, case‐control, and cohort) reporting data on salivary chemical elements. Both prospective and retrospective studies were included.

#### 2.2.2 Control and target condition

The target conditions were chronic (CP) and aggressive (AgP) forms of P, regardless of severity and extent, according to the classification of Armitage^17^ or P of any stage and grade according to the current classification.^18^ As controls, G and H conditions were considered. The diagnosis was considered both at patient and site level. Only studies comparing P patients versus H or G subjects were selected.

#### 2.2.3 Exclusion criteria

Studies investigating salivary ionic profiling in specific medical conditions (i.e., diabetes), as well as studies evaluating elemental changes after periodontal treatment, were excluded. In vitro studies, animal studies, editorials, clinical case reports, and literature reviews were not included, as well as articles not written in English.

### 2.3 Search methods for the identification of studies

Reviewers were calibrated for study screening, data extraction, and risk of bias assessment against another experienced reviewer. Literature searches were performed on three databases (Medline via PubMed, Embase, and Scopus) in duplicate by 2 reviewers. Studies were collected between January 1, 2010, and May, 31 2021. In addition, the references of all included studies and relevant reviews were manually cross‐checked to ensure complete data collection. The detailed search strategy is reported in Appendix S1.

### 2.4 Study selection

Titles and abstracts were screened independently by two reviewers (G.I. and M.C.), and full text of potentially eligible studies was obtained for independent assessment by the same reviewers. Any disagreement was solved by discussion until consensus was reached. Where resolution was not possible, a third reviewer was consulted (G.B.). The reasons for exclusion of studies after full‐text analysis were recorded. The inter‐reviewer reliability (percentage of agreement and kappa correlation coefficient) of the screening method was calculated.

### 2.5 Data extraction and management

Data were extracted by two authors independently using specially designed templates (G.I. and M.C.). In case of a prospective clinical study, only data recorded before treatment were collected. Similarly, in case of a study comparing periodontal status in patients with and without specific medical conditions, only data of systemically healthy individuals were extracted. Disagreement was resolved through discussion with a third reviewer (G.B.).

### 2.6 Risk of bias in the included studies and quality assessment

The quality assessment of the included studies was carried out independently and in duplicate by two reviewers (G.I. and M.C.) using a modified version of the NIH Quality Assessment Tool for Observational Cohort and Cross‐Sectional Studies and the QUADOMICS tool, which has been developed for omics research.^9^, ^19^ This tool includes 13 questions specifically addressed to evaluate the research question, study population, exposure, outcomes, and statistics in omics studies. Every item was given either 1 or 0 point, and, consequently, studies were rated based on their total score, where 0 < 3 was considered very low, 4 < 6 as low, 7 < 11 as moderate, and ≥11 as high.

### 2.7 Strategy for data synthesis

Element concentration was originally reported as mean ± standard deviation (SD), median (interquartile range, IQR), or median (min–max). Effort was made to convert the level of each chemical element to mean ± SD using the same unit of measure to allow comparisons. Where data were reported as median (IQR) or median (min–max), the correspondent mean and SD were calculated according to the method described by Hozo et al.^20^ Due to very high heterogeneity in methodologies and outcomes among the included studies, meta‐analyses of data were attempted but did not deserve publication. However, a consistency analysis was presented focusing on chemical elements that were significantly discriminant between P and H groups.

## 3 RESULTS

### 3.1 Study selection

**FIGURE A1:**
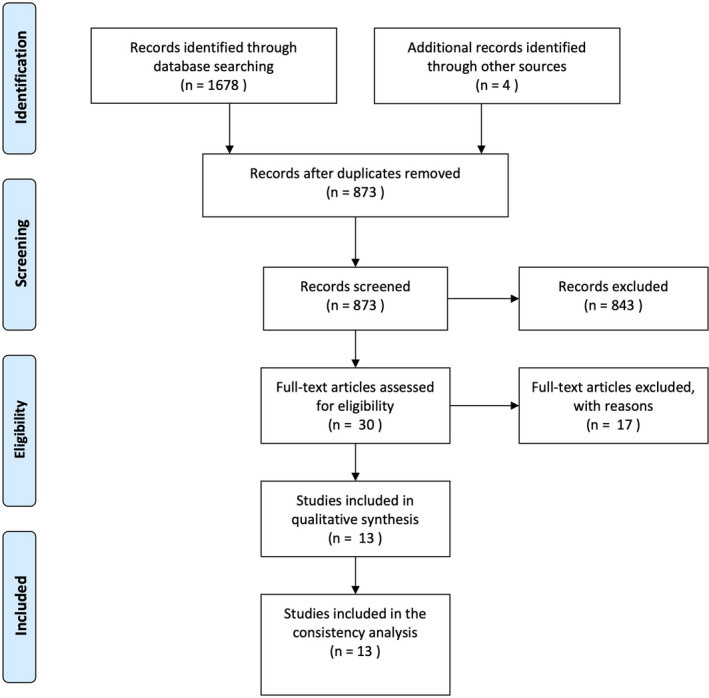
PRISMA flowchart of the selection process

Figure A1 depicts the study flow chart. In total, 869 documents were obtained from the electronic search after removal of duplicates and 4 additional articles were identified from the manual search. After the title and abstract evaluation process, 30 articles were analyzed in full text. Of these, 17 articles were excluded for various reasons (Appendix S2), and 13 papers were selected for the qualitative analysis.^21^, ^22^, ^23^, ^24^, ^25^, ^26^, ^27^, ^28^, ^29^, ^30^, ^31^, ^32^, ^33^ The measure of inter‐reviewer agreement was k = 0.87 for abstract screening and k = 0.93 for full‐text analysis.

### 3.2 Study characteristics

Appendix S3 summarizes the main characteristics of the included studies. The majority of the studies enrolled Asian populations. Five studies were conducted in India, 2 in Iraq, while the others were from Turkey, Italy, Croatia, Poland, and Romania. The number of participants ranged from 30 to 190, with P patients ranging from 15 to 90 and H controls ranging from 14 to 50. The age ranged from 25 to 60 years for both groups, although a tendency for a younger age was encountered in aggressive P patients and controls. Most of the studies had a cross‐sectional design. Three studies performed a broad spectrum ionomic analysis to maximize element discovery (untargeted analysis),^21^, ^22^, ^31^ while the remaining 10 studies aimed at identifying specific trace elements (targeted analysis). Periodontitis case definitions and periodontal parameters were quite heterogeneous across the studies. All studies enrolled patients with CP, while only one article included patients with AgP. Only 2 studies included G as control group. Smoking habit was an exclusion criterion in most studies.

**TABLE 1:** Methods of saliva collection and sample analysis across the included studies

Authors	Pre‐sampling procedures		Saliva collection method			Pre‐analytical procedures		Detection method
	Restrictions	Timing	Type of saliva	Volume	Time of sampling	Sample storage	Sample preparation	
Inonu et al. (2020)	No eating	1 h before	Unstimulated saliva	3 ml	NR	−80 °C	Yes	Untargeted: ICP‐MS
Romano et al. (2020)	No alcohol, food, sugar drinks, caffeine, toothpastes and mouthwashes	The morning of the collection 12 h before (alcohol)	Unstimulated saliva	5 ml	8–10 am	−80 °C	Yes	Untargeted: SF‐ICP‐MS equipped with a glass concentric nebulizer and a Twinnabar (cyclonic) spray chamber
Santo Grace et al. (2019)	NR	NR	Unstimulated saliva	NR	NR	NR	NR	Targeted: Digital analyser
Karwasra et al. (2018)	No eating, drinking, tooth brushing and smoking	1 h before	Unstimulated saliva (water rinsing 1 min before collection)	2 ml	NR	NR	NR	Targeted: OCPC method in a semi‐automated Erba Mannheim CHEM−5 Plus v2 machine
TalalAbd et al. (2017)	NR	NR	Unstimulated saliva (water rinsing 1–2 mins before collection)	5 ml	Early in the morning	−80 °C	Only centrifuge	Targeted: Flame Atomic Absorption Spectrophotometer
Natarajan et al. (2016)	No eating	2 h before	NR (distilled water rinsing before collection)	5 ml	NR	4 °C	Only centrifuge	Targeted: Colorimetric method
Patel et al. (2016)	No eating	2 h before	Unstimulated saliva (distilled water rinsing before collection)	5 ml	10–11 am	2–4 °C	Only centrifuge	Targeted: Arsenazo reagent (Ca) Molybdate U.V. method (P)
Boras et al. (2016)	NR	NR	Unstimulated saliva	0.2 ml	9–12 am	−20 °C	NR	Targeted: Indirect potentiometry ‐ Roche Cobas C51 (Na, K, Cl) AAS (Cu)
Herman et al. (2016)	NR	NR	Unstimulated saliva	3 ml	In the morning	−80 °C	Only centrifuge	Targeted: ICP‐OES (Ca, Mg) ICP‐MS (Mn, Fe, Cu, Zn, Cd and Pb)
Manea and Nechifor (2014)	No eating, drinking and oral hygiene	The morning of the collection	NR	NR	In the morning	Desiccation at 400 °C for 24 hours	Yes	Targeted: AAS
Huang et al. (2014)	No eating and drinking	At least 30 min before	Unstimulated saliva (water rinsing before collection)	NR	8–12 am	−80 °C	Only centrifuge	Untargeted: ICP‐MS
Abid Aun et al. (2012)	No eating and drinking (excluding water)	At least 1 h before	Unstimulated saliva	5 ml	9–12 am	−20 °C	Only centrifuge	Targeted: Flame AAS
Acharya et al.(2011)	No eating and drinking	At least 1 h before	Unstimulated saliva	3 ml	In the morning	NR	NR	Targeted: Calcium ion selective electrode method with a AVL9180 ion selective electrolyte analyser

Abbreviations: AAS, atomic absorption spectrophotometer; ICP‐MS, inductively coupled plasma mass spectrometer; ICP‐OES, inductively coupled plasma optical emission spectroscopy; NR, not reported; OCPC, o‐cresolphthalein complexone; SF‐ICP‐MS, sector field inductively coupled plasma mass spectrometry.

Details about saliva collection and analytical protocols are summarized in Table 1 . Three studies did not report any information on pre‐sampling procedures, and only 4 studies detailed the hour of sample collection. All studies collected unstimulated whole saliva samples in different volumes. Inductively coupled plasma mass spectrometry (ICP‐MS) was the method most reported in literature for untargeted ion determination. For targeted analysis, the detection methods were strictly dependent on the chemical element analyzed. Atomic absorption spectrophotometer (AAS) using standardized procedure by air acetylene was used for the determination of calcium (Ca), magnesium (Mg), potassium (K), sodium (Na),^32^ and copper (Cu).^28^ Indirect potentiometry was used for the determination of Na, K, and chloride (Cl),^28^ while ion selective electrodes (ISE) were also used for the determination of Ca.^33^

**TABLE 2:** Overview of the main findings of the included studies

Authors (year)	Sample size (Males/Females)		Periodontal status at baseline		Metal concentrations	
	Case	Control	Chronic Periodontitis (%)	Healthy (%)	Chronic Periodontitis [mean ±SD]	Healthy [mean ±SD]
Inonu et al. (2020)	50 (26/24)	50 (19/31)	50 (26.3)	50 (26.3)	**Ca: 79.4 ± 30.83** **Cr: 0.11 ± 0.06** **Fe: 14.76 ± 13.73** **Mg: 14.8 ± 6.5** **Mn: 484 ± 25** **Na: 447.5 ± 223.5** **K: 5448 ± 4430** **Rb: 0.78 ± 0.22** **Se: 0.06 ± 0.02** Sr: 7.99 ± 8 **V: 0.02 ± 0.01** Zn: 3638.5 ± 3462	Ca: 48.75 ± 21.78 Cr: 0.11 ± 0.06 Fe: 4.72 ± 8.5 Mg: 8.23 ± 3.78 Mn: 197 ± 146 Na: 223 ± 119.5 K: 852.7 ± 240.7 Rb: 0.78 ± 0.22 Se: 0.03 ± 0.03 Sr: 0.69 ± 0.67 V: 0.02 ± 0.01 Zn: 2359.8 ± 2210
Romano et al. (2020)	24 (17/7)	26 (12/8)	24 (54.5)	20 (45.5%)	Ba: 1.57 ± 0.12 Ca: 28.33 ± 13.42 Cu: 31.58 ± 15.03 **Fe: 26.66 ± 5.62** K: 967.0 ± 66.30 Li: 1.19 ± 0.21 Mg: 7.67 ± 1.013 **Mn: 7.29 ± 1.15** **Na: 289.70 ± 36.06** Zn: 55.45 ± 11.12	Ba: 1.13 ± 0.15 Ca: 30.05 ± 10.06 Cu: 11.95 ± 1.86 Fe: 9.74 ± 1.47 K: 1025.27 ± 61.59 Li: 0.99 ± 0.17 Mg: 6.31 ± 0.59 Mn: 3.52 ± 0.58 Na: 152.07 ± 19.41 Zn: 46.01 ± 6.28
Santo Grace et al. (2019)	NR	NR	15 (50.0)	15 (50.0)	**Cu: 88.14 ± 2.09** **Zn: 63.04 ± 2.31**	Cu: 74.97 ± 1.23 Zn: 72.78 ± 2.83
Karwasra et al. (2018)	30 (30/0)	30 (30/0)	30 (50.0)	30 (50.0)	Smokers: 80.87 ± 1.70 **Non‐smokers: 80.65 ± 2.69**	Smokers: 80.69 ± 1.68 Non‐smokers: 70.32 ± 1.70
TalalAbd et al. (2017)	25 (NR)	15 (NR)	25 (62.5)	15 (37.5)	**Cd: 1.79 ± 0.16** Mg: 5.15 ± 0.73 **Pb: 54.00 ± 15.68**	Cd: 1.09 ± 0.25 **Mg: 10.24 ± 3.49** Pb: 20.87 ± 3.002
Natarajan et al. (2016)	15 (NR)	15 (NR)	15 (50.0)	15 (50.0)	**Na: 803 ± 174** **Cl: 52600 ± 13814**	**Na: 240.5 ± 114.5** **Cl: 24866.7 ± 8348.37**
Patel et al. (2016)	50 (18/32)	50 (22/28)	50 (33.3)	50 (33.3)	**Ca: 1255 ± 273** **P: 14.50 ± 3.82**	Ca: 547 ± 139 P: 3.93 ± 1.89
Boras et al. (2016)					Cl: 32.1656 ± 11.10250 **Cu: 24.84 ± 8.89** K: 899346 ± 274417 Na: 318 ± 53.6	Cl: 34.4512 ± 13.03582 Cu: 18.68 ± 10.64 K: 918427 ± 277037 Na: 294 ± 93.1
Herman et al. (2016)	31 (14/17)	29 (10/19)	31 (51.7)	29 (48.3)	Ca: 39.2 ± 19.4 Cd: 0.2 ± 0.1 **Cu: 45.1 ± 5.0** Fe: 1.0 ± 0.6 **Mg: 9.9 ± 5.4** **Mn: 41.1 ± 15.6** Pb: 15.8 ± 8.2 Zn: 79.1 ± 103.2	Ca: 35.0 ± 18.4 Cd: 0.3 ± 0.3 Cu: 8.2 ± 5.2 Fe: 0.9 ± 0.7 Mg: 5.2 ± 2.5 Mn: 15.0 ± 8.2 Pb: 12.4 ± 7.9 Zn: 75.3 ± 74.4
Manea and Nechifor (2014)	12 (11/1)	9 (7/2)	12 (57.1)	9 (42.9)	Ca: 53.37 ± 6.57 **Mg: 1.07 ± 0.18** **Cu: 1200 ± 200** Zn: 370 ± 10	Ca: 84.24 ± 0.87 Mg: 0.81 ± 0.001 Cu: 830 ± 180 Zn: 480 ± 50
Huang et al. (2014)	50 (21/29)	50 (21/29)	50 (50.0)	50 (50.0)	Al: 6.83 ± 0.65 Ca: 1.07 ± 0.44 Cd: 0.04 ± 0.07 Cs: 0.34 ± 0.02) Cu: 2.67 ± 1.49 Fe: 0.025 ± 0.001 K: 118.8 ± 42.5 Mg: 0.93 ± 0.43 Mn: 2.89 ± 2.33 Ni: 0.71 ± 0.07 Sr: 3.82 ± 0.23 Pb: 0.33 ± 0.21 Rb: 0.14 ± 0.01 Zn: 24.83 ± 12.3	**Al: 15.43 ± 1.45** **Ca: 1.34 ± 0.59** **Cd: 0.04 ± 0.07** **Cs: 0.42 ± 0.02** **Cu: 3.36 ± 1.84** **Fe: 0.024 ± 0.009** **K: 165.6 ± 45.5** **Mg: 1.46 ± 0.83** **Mn: 12.17 ± 9.83** Ni: 0.87 ± 0.1 **Sr: 11.53 ± 1.65** **Pb: 0.51 ± 0.35** **Rb: 0.18 ± 0.01** **Zn: 30.93 ± 18.1**
Abid Aun et al. (2012)	30 (18/12)	30 (15/15)	30 (50.0)	30 (50.0)	**Ca: 77.8 ± 16.8** **K: 342.1 ± 53.6** Mg: 6.6 ± 1.5 **Na: 394.5 ± 61.8**	Ca: 49.3 ± 12 K: 288.2 ± 33.6 **Mg: 7.3 ± 1.7** Na: 274.3 ± 27.1
Acharya et al. (2011)	25 (15/10)	25 (15/10)	25 (50.0)	25 (50.0)	**Ca: 84.6 ± 9.6**	Ca: 74.5 ± 10

Legend: Ca, Cr, Fe, K, Mg, Na, Rb, Se, and V concentrations were expressed in mg/L; Al, Ba, Cd, Cs, Cu, Li, Mn, Ni, P, Pb, Sr, and Zn concentrations in μg/L; while Cl concentrations in μmol/L. Statistically significant upregulation of elements was represented in bold.

Table 2 summarizes in detail the results of the included studies. A total of 22 elements were analyzed across the 13 studies, including aluminum, barium, Ca, cadmium (Cd), Cl, chromium, cesium, Cu, iron (Fe), K, lithium (Li), Mg, manganese (Mn), Na, nickel (Ni), selenium (Se), strontium (Sr), phosphorus, lead (Pb), rubidium, vanadium, and zinc (Zn). Ca was the most investigated element, recurring in 9 studies, followed by Mg (8 studies), Cu and Zn in 6 studies each, Na and K in 5 studies, Fe and Mn in 4 studies, while Cd and Pb in 3 articles. No studies reported about measures of diagnostic accuracy.

### 3.3 Quality assessment

Appendix S4 reports the results of the methodological quality assessment using the QUADOMICS tool. While the almost totality of the studies adopted well‐defined clinical criteria for P, G, and H subject selection (Items 2 to 7), operative protocols and reporting were less rigorous for saliva sampling methods and statistical analysis. Four studies were considered as high quality, 5 studies as moderate quality, while the others were rated as low or very low quality.

### 3.4 Altered elemental profiles in periodontitis and healthy individuals

**FIGURE 1:**
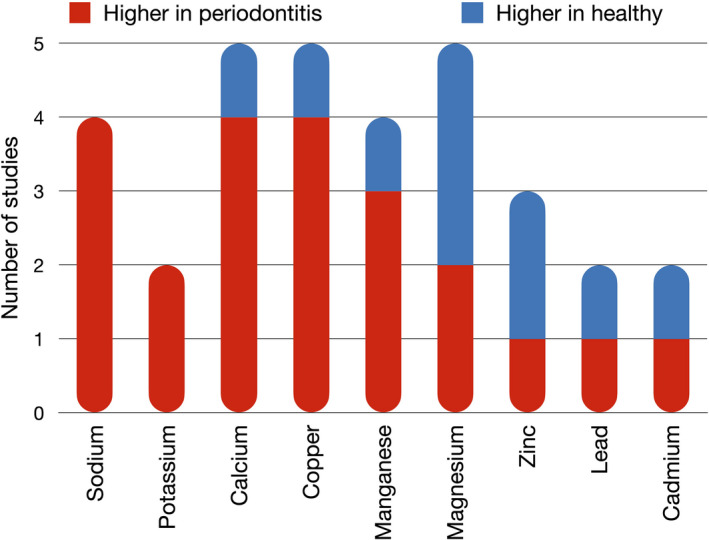
Consistency analysis for the differentially quantified ions in saliva of patients with periodontitis compared to individuals with periodontal health. Only statistically significant changes were included

Figure 1 illustrated the results from the consistency analysis. Sodium and potassium revealed a consistent tendency to increase in P patients compared to H in 3 and 2 different studies, respectively. Na levels ranged from 289 to 803 mg/L in P and from 158 to 294 mg/L in H, while K values ranged from 119 to 899 346 mg/L in P and from 166 to 918 427 mg/L in H.

For all the other elements, conflicting results were obtained. For calcium and copper, the majority of studies recorded significant increases in P (4 studies), while only one reported an inverse tendency. Ca values ranged from 1 to 1255 mg/L in P group and from 1 to 547 mg/L in H group, while Cu values ranged from 3 to 1200 μg/L in P and from 3 to 830 μg/L in H. Ca levels were found significantly increased in smokers irrespectively of the clinical diagnosis of P.^24^ Manganese concentration was found significantly higher in saliva of P in 3 studies, while lower in the study by Huang et al.^31^ Values for Mn ranged from 3 to 484 μg/L in CP and from 4 to 197 μg/L in H. Controversial results were available for magnesium, whose levels were found higher in P than in H in 2 studies and lower in 3 studies. Mg values ranged from 1 to 15 mg/L and from 1 to 10 mg/L in H controls. Zinc resulted significantly increased in saliva from H individuals compared to P in two studies, but one study reported opposite findings. Zn values ranged from 25 to 3638 μg/L in P and from 31 to 2360 μg/L in H. Finally, levels of cadmium and lead resulted significantly increased in P in one study, but decreased in another experiment. Cd concentrations varied from 0.04 to 1.79 μg/L in P and from 0.04 to 1.09 μg/L in H, while Pb values ranged from 0.33 to 54 μg/L in P and from 0.51 to 20.87 μg/L in H. The effect of the periodontal disease emphasizes the increase in Fe concentrations (0.513–0.775 g/L) comparatively to H.

The high heterogeneity in the outcome measures for each chemical element did not render meta‐analyses reliable, and therefore, they were not presented.

## 4 DISCUSSION

Macro and trace minerals are functional to periodontal maintenance, and their alteration in biofluids can represent a sign of pathological conditions.^34^ Our study was the first systematic review to evaluate the salivary ionic profile of subjects with different periodontal statuses. Results for 22 specific elements were sorted from 13 included reports. Consistent outcome measures were found for those elements related to the pathways of immune‐modulation and bone tissue homeostasis.

### 4.1 Outcomes for specific metals

Periodontitis results as the consequence of a chronic immune‐inflammatory reaction to the microbial challenge that brings to the destruction of tooth‐supporting apparatus and to the discharge of inflammatory markers and breakdown products in gingival crevicular fluid (GCF) and saliva.^5^, ^35^ Therefore, it is rationale to hypothesize that perturbations in ionic content of these biofluids would be suggestive of the presence and severity of periodontitis.

Na concentrations were found consistently higher in saliva of patients with chronic or aggressive periodontitis with respect to individuals with periodontal health. Na represents one of the most abundant elements in humans, mainly distributed in blood, bone, and connective tissues where it mainly set the balance of fluids and nutrients throughout the cellular membrane.^36^ Following the destruction of the alveolar bone tissue, Na can be released into the extracellular compartment and hence into the GCF and saliva.^19^, ^37^ Some authors have reported that Na levels tend to increase proportionally according to the severity of the clinical attachment level loss and bleeding,^38^, ^39^ while others failed to confirm it.^32^ An antibacterial function of salivary Na ions has also been recognized, possibly suggesting its elevation in saliva as a mechanism of defense.^26^

Increased salivary Ca concentrations have been widely related to periodontitis in the present review. Several biological processes may be accounted for this shift. Calcium is an essential mediator for intracellular signaling pathways, which during the development of periodontal inflammation is upregulated to stimulate reactive oxygen species (ROS) production and the expression of cytokine mediators, such as tumor necrosis factor‐alpha (TNF‐α).^40^ Therefore, both intracellular calcium increment and hard tissue breakdown could contribute to the elevated concentration of salivary calcium. Four authors showed a strong significant difference in the salivary levels of ions between healthy and periodontal subjects, with a higher concentration in P.^21^, ^27^, ^32^, ^33^ Increased levels of this metal in saliva influence the mineralization of dental plaque and therefore calculus formation, which is a risk factor for the development of gingivitis that can later evolve into periodontitis.^41^ The authors also found a significant correlation between Na, Ca, and clinical attachment levels, supporting a possible dose‐dependent effect between ion imbalance and the severity of the disease.

Increased Cu levels were also associated with periodontal breakdown. It is known how Cu and Zn functions can be closely related, being key constituents of antioxidant enzymes like Cu–Zn superoxide dismutase (SOD).^42^ The results from the present review indicate that there is a tendency for an excess of Cu and Zn decrease within the saliva of periodontitis patients. This can be due to the fact that elevated levels of Cu can alter the permeability of the gingival epithelium and impair Zn mucosal absorption.^43^ Notably, Zn concentration was restored to physiologic levels after successful periodontal treatment.^22^ Moreover, salivary histatins are relevant antimicrobial enzymes within the oral cavity and they are composed of copper.^44^ Therefore, increased salivary copper levels might indicate that either SOD and/or histatins are not well functioning. Accordingly, histatin 3 gene was revealed under expressed in gingival smears of P compared to H, possibly indicating a host defense dysregulation.^45^

Among the controversies, magnesium was the element which was examined in most studies. Three of them reported a decreased Mg concentration in the saliva of P. Notably, a close association between Mg deficiency and macrophage and leukocyte overactivation has been demonstrated across multiple conditions, as Mg can elicit oxidative‐inflammatory processes by impairing intracellular Ca.^46^ Conversely, Mg is abundantly represented in calcified tissues, and when periodontal breakdown occurs, the release of Mg can produce an increase in its salivary concentration, even in presence of Mg deficiency.^47^ As for other ions, some authors found a direct correlation between salivary Mg levels and severity of periodontal diseases observing an increase in salivary Mg levels in patients with gingivitis. Magnesium can also have a negative influence on intracellular and salivary K concentration. This was suggested by Kaslick et al.^48^ and Aun^32^ which found an enhanced salivary K concentration in P associated with magnesium deficiencies, although further studies are required to provide mechanistic explanation.

Notably, the study by Huang et al.^31^ reported outcomes in marked contrast with the rest of the included works, despite using the same technique (ICP‐MS) and comparable case definitions. The authors found a higher level of all analyzed chemical elements in the unstimulated saliva of healthy subjects compared to P. These discrepancies can be attributed to ethnic aspects related to genetic features and dietary habits, being the level of these micronutrients strictly associated with the food frequency questionnaire of the population analyzed.

More than per se variations, it would be relevant to understand whether metal ion concentration could serve for diagnostic purposes. To this regard, only two studies implemented ionomic analysis into discriminating models. Romano et al.^22^ developed a cluster analysis which was able to correctly separate active periodontitis and periodontally healthy individuals, whereas treated periodontitis individuals were classified as H. Also, Herman et al.^29^ successfully applied such a statistical approach to distinguish CP from H.

### 4.2 Methodologic issues

In the last years, ionomics has received an impressive development owing to the implementation of reliable measurement technologies and instruments for large‐scale data analysis. Mineral element levels in saliva have been traditionally measured by different methods, such as AAS, ICP‐optical emission spectrometry (OES), potentiometry, thermal neutron activation analysis, and gamma ray spectrometry. In particular, the most commonly used in ionomics is ICP‐MS, because it allows fast and accurate routine multi‐element determination with improved sensitivity for biological samples.^49^ Considering the high heterogeneity of findings for each metal concentration, the employment of different detection method could be a major determinant.

Also, circadian and seasonal variations are of utmost importance when interpreting the findings from metabolites and ions in saliva, as well as for the effect of tobacco smoking.^50^ Standardization of sampling procedures, in accordance to dietary restrictions, home oral hygiene restraints, and timing of collection are of primary importance.^19^

A thorough assessment of periodontal status represents the reference standard for periodontal diagnosis; nonetheless, only few studies accurately reported how periodontal parameters were recorded and the definition of periodontal cases largely varied across the included studies. It has been shown that patients with untreated periodontitis had values above those of H and patients with restored healthy periodontium at the completion of active periodontal therapy, while levels of treated periodontitis patients were not statistically different from those of H.^22^ Ethnic background and variations in sample sizes can also account for a large part of the variability.^51^, ^52^ Finally, most of the included studies did not get high scores in the quality assessment.

### 4.3 Future research directions

Despite ionomics being rapidly developing in different fields of medicine through the last years, our understanding of the relationship between different ions and the pathogenesis of complex diseases is still limited. This review critically examined the available evidence on ionomics as a new platform to improve early diagnosis, prognosis evaluation, and therapy of periodontal diseases. Meaningful reduction and integration of big data arising from ion‐based high‐throughput techniques is highly demanded to find the signal among the noise and to successfully translate biochemical signatures into clinically relevant information. Whether significant differences in macro and trace elements between patients will become evident, a microfluidic chair‐side test could be developed in both early diagnosis and/or monitoring of the clinical progression/response to the treatment. To this regard, only one study considered the elemental profiles of successfully treated periodontitis patients.

From the present systematic review, ionomics emerges as a promising landscape for biomarkers discovery and implementation for diagnostic purposes applied to periodontology, despite its preliminary results could be hampered by environmental and technical biases in elemental composition and detection. Sodium and potassium were the elements found consistently and significantly higher in saliva of P compared to H. Due to high concerns in developing more accurate, non‐invasive, and portable diagnostic tools for individual use and population‐based strategies, the results from the present work indicate a great need to identify and quantify macro and trace elements in saliva for periodontitis by providing measures of diagnostic accuracy.

## CONFLICTS OF INTEREST

The authors declare that they have no competing interests.

## AUTHOR CONTRIBUTIONS

GB, GI, GNB, and MA made substantial contributions to conception of the study. GB, GI, MC, FC, FR, and MA contributed to the study design. GB, GI, and MC searched and collected the data. GB, FR, AG, and FR performed data analysis and interpretation. GB, FR, AG, and GNB prepared the first draft of the manuscript. All authors have read, revised critically, and approved the final manuscript.

## Supporting information

Appendix S1

Appendix S2

Appendix S3

Appendix S4

## Data Availability

All data generated or analyzed during this study are included in this published article [and its supplementary information files].
