# Supplementary material for: Macro and trace elements signature of periodontitis in saliva: A systematic review with quality assessment of ionomics studies
Source: J Periodontal Res. 2021 Nov 27;57(1):30–40. doi: 10.1111/jre.12956 (PMC9298699; doi:10.1111/jre.12956)
Supplement: Supplementary file 1 — Appendix S1 [file JRE-57-30-s003.docx]

**Appendix S1**

**Search strategy**

**Search 1: [MEDLINE ] (via PUBMED)**

(ion OR ionomic OR ionic OR sodium OR potassium OR calcium OR magnesium OR fluoride OR iron OR phosphate OR sulphite OR manganese OR zinc) AND (saliva OR salivary OR GCF OR crevicular) AND (periodontitis OR periodontal disease)

**Search 2: [ EMBASE ]**

('ion'/exp OR ion OR ionomic OR ionic OR 'sodium'/exp OR sodium OR 'potassium'/exp OR potassium OR 'calcium'/exp OR calcium OR 'magnesium'/exp OR magnesium OR 'fluoride'/exp OR fluoride OR 'iron'/exp OR iron OR 'phosphate'/exp OR phosphate OR 'sulphite'/exp OR sulphite OR 'manganese'/exp OR manganese OR 'zinc'/exp OR zinc) AND ('saliva'/exp OR saliva OR salivary OR gcf OR crevicular) AND ('periodontitis'/exp OR periodontitis OR 'periodontal disease'/exp OR 'periodontal disease' OR (periodontal AND ('disease'/exp OR disease)))

**Search 3: [ SCOPUS ]**

(ionomic OR ionic OR sodium OR potassium OR calcium OR magnesium OR fluoride OR iron OR phosphate OR sulphite OR manganese OR zinc) AND periodontitis AND saliva
