# Supplementary material for: Macro and trace elements signature of periodontitis in saliva: A systematic review with quality assessment of ionomics studies
Source: J Periodontal Res. 2021 Nov 27;57(1):30–40. doi: 10.1111/jre.12956 (PMC9298699; doi:10.1111/jre.12956)
Supplement: Supplementary file 2 — Appendix S2 [file JRE-57-30-s001.docx]

**Appendix S2.** Studies excluded after full text reading, with reason for exclusion.

**Reasons for exclusion (*Legend*)**

| Reference | Reason For Exclusion |
| --- | --- |
| Toczewska et al. (2020) | 1 |
| de Castro et al. (2019) | 1 |
| Grupta et al. (2016) | 2 |
| Prashaanthi et al. (2016) | 2 |
| Moghadam et al. (2016) | 3 |
| Varghese et al. (2015) | 3 |
| de Medeiros et al. (2015) | 2 |
| Meschiari et al. (2015) | 1 |
| Sanchez et al. (2014) | 1 |
| Fiyaz et al. (2013) | 2 |
| Lewgoy et al. (2013) | 4 |
| Han et al. (2013) | 1 |
| Sharath et al. (2013) | 5 |
| Zhang et al. (2013) | 5 |
| Mahmood et al. (2012) | 2 |
| Sutej et al. (2012) | 3 |
| Kiss et al. (2010) | 5 |

1. Oxidative stress markers
2. No units of measure
3. No healthy control group
4. No ion concentration
5. No full text available
