# Supplementary material for: Macro and trace elements signature of periodontitis in saliva: A systematic review with quality assessment of ionomics studies
Source: J Periodontal Res. 2021 Nov 27;57(1):30–40. doi: 10.1111/jre.12956 (PMC9298699; doi:10.1111/jre.12956)
Supplement: Supplementary file 3 — Appendix S3 [file JRE-57-30-s004.docx]

**Appendix S3** Characteristics of studies and participants in the systematic review

|  |  |  | **Smokers** | | **Age (years) Mean ± SD or Mean (Range)** | | **Periodontal definition selected** | | **Periodontal status at baseline** | |  |
| --- | --- | --- | --- | --- | --- | --- | --- | --- | --- | --- | --- |
| **Authors (year)** | **Region** | **Study design** | **Case** | **Control** | **Case** | **Control** | **Case** | **Control** | **Case (%)** | **Control (%)** | **Periodontal status assessment** |
| Inonu et al. (2020) | Konya, Turkey | Cross-sectional | 0 | 0 | G: 24.5 (20.0-56.0)  CP: 43.5 (27.0-58.0)  GAgP: 28.0 (20.0-35.0) | 28.0 (25.0-41.0) | **G:** GI > 0, PPD ≤3 mm, and CAL ≤2 mm at ≥90% of teeth; no radiographic signs of ABL  **CP:** CAL ≥3 mm, GI >0, and PPD ≥6 mm at multiple sites  **GAgP:** rapid attachment loss and bone destruction inconsistent with amounts of microbial deposits; <30 years of age | **H:** PPD ≤ 3 mm, no more than 10% of the sites with BoP; absence of gingival redness/edema and radiographic evidence of ABL | **G:** 50 (26.3)  **CP:** 50 (26.3)  **GAgP:** 40 (21.1) | 50 (26.3) | Full-mouth |
| Romano et al. (2020) | Turin, Italy | Cross-sectional | 0 | 0 | 55.4 ± 13.8 | 44.3 ± 10.8 | Untreated generalized periodontitis stage III or IV grade B or C | No evidence of periodontal disease; all PDs ≤3 mm, FMBS <10%; no radiographic evidence of ABL. | 24 (54.5) | 20 (45.5%) | Full-mouth |
| Santo Grace et al. (2019) | Chennai, India | Prospective | NR | NR | NR | NR | **CP:** diagnosis according to the American Academy of Periodontology criteria (1999); PPD ≥4 mm | NR | 15 (50.0) | 15 (50.0) | NR |
| Karwasra et al. (2018) | Rajasthan, India | Cross-sectional | 30 (50%) | 30  (50%) | 25-55 (overall range) | 25-55 (overall range) | **CP:** diagnosis according to the PSR | NR | 30 (50.0) | 30 (50.0) | NR |
| TalalAbd et al. (2017) | Baghdad, Iraq | Cross-sectional | NR | NR | 25-45 (overall range) | 25-45 (overall range) | **CP** diagnosis based on GI and CAL | Healthy gingiva based on GI and CAL | 25 (62.5) | 15 (37.5) | NR |
| Natarajan et al. (2016) | Chennai, India | Prospective | NR | NR | NR | NR | **CP** no further specified | NR | 15 (50.0) | 15 (50.0) | NR |
| Patel et al. (2016) | Maharashtra, India | Cross-sectional | NR | NR | G: 29.59 ± 5.47  P: 41.96 ± 2.44 | 24.94 ± 2.53 | **G:** PPD ≤3 mm with no LOA; presence of BOP  **P:** PPD >4 mm and CAL loss with or without BoP. | PPD ≤3 mm, no attachment loss and BoP | G: 50 (33.3)  P: 50 (33.3) | 50 (33.3) | Full-mouth |
| Boras et al. (2016) | Zagreb, Croatia | Cross-sectional | 59 | 17 | 45.3 ± 11.6 | 47.3 ± 21.1 | **CP:** diagnosis according to the American Academy of Periodontology criteria (1999) | No sites with PPD >3 mm, no radiographic bone loss, and no BoP on >2 teeth | 35 (46.1) | 41 (53.9) | Full-mouth |
| Herman et al. (2016) | Krakow, Poland | Comparative study | 0 | 0 | 34.5 ± 10.4 | 31.8 ± 16.8 | **P** according to the CPITN | **H** according to CPITN | 31 (51.7) | 29 (48.3) | NR |
| Manea et al. (2014) | Iași, Romania | Comparative study | 21 | 39 |  |  | **P:** loss of gingival-dental attachment; PPD >3 mm; radiological loss of interdental septa (horizontal bone loss) or of lamina dura (vertical bone loss) | NR | 12 (57.1) | 9 (42.9) | Full-mouth |
| Huang et al. (2014) | Shangha, China | Cross-sectional | 100 | 0 | 46.40 (±7.62) | 46.22 (±7.28) | 2 or 3 nonadjacent PPD ≥ 4 mm or CAL ≥3–4 mm; presence of BoP | **H**: good oral hygiene with no clinical signs of gingival inflammation, deep pockets, or tooth mobility. | 50 (50.0) | 50 (50.0) | Full-mouth |
| Abid Aun et al. (2012) | Baghdad, Iraq | Comparative study | 0 | 0 | 30-50 (overall range) | 30-50 (overall range) | **CP**: PPD ≥ 4 mm with CAL loss | No PPD, BoP and bone loss | 30 (50.0) | 30 (50.0) | Full-mouth |
| Acharya et al. (2011) | Pimpri, India | Comparative study | 0 | 0 |  |  | **CP:** ≥30% of sites with LOA and FMBS ≥10% | No LOA, FMBS <10% | 25 (50.0) | 25 (50.0) | Full-mouth |

Abbreviations: ABL, alveolar bone level; BoP, bleeding on probing; CAL, clinical attachment level; CP, chronic periodontitis; CPITN, community periodontal index of treatment needs; FMBS, full-mouth bleeding score; G, gingivitis; GAgP, generalized aggressive periodontitis; GI, gingival index; H, periodontally healthy individuals; LOA, loss of attachment; NR, not reported; P, periodontitis; PPD, probing pocket depth; PSR, periodontal screening and recording.
