# Supplementary material for: Macro and trace elements signature of periodontitis in saliva: A systematic review with quality assessment of ionomics studies
Source: J Periodontal Res. 2021 Nov 27;57(1):30–40. doi: 10.1111/jre.12956 (PMC9298699; doi:10.1111/jre.12956)
Supplement: Supplementary file 4 — Appendix S4 [file JRE-57-30-s002.docx]

**Appendix S4** Quality assessment of the included studies evaluated by a modified version of the QUADOMICS tool

| **Item** | Inonu et al. (2020) | Romano et al. (2020) | Santo Grace et al. (2019) | Karwasra et al. (2018) | TalalAbd et al. (2017) | Natarajan et al. (2016) | Patel et al. (2016) | Boras et al. (2016) | Herman et al. (2016) | Manea et al. (2014) | Huang et al. (2014) | Abid Aun et al. (2012) | Acharya et al. (2011) |
| --- | --- | --- | --- | --- | --- | --- | --- | --- | --- | --- | --- | --- | --- |
| 1. Was the research question or objective in this paper clearly stated and appropriate? | Yes | Yes | Yes | Yes | Yes | Yes | Yes | Yes | Yes | Yes | Yes | Yes | Yes |
| 2. Was the study population clearly specified and defined? | Yes | Yes | No | No | Yes | No | Yes | Yes | Yes | Yes | Yes | Yes | Yes |
| 3. Were procedures and timing of biological sample collection with respect to clinical factors described with enough detail? | 0.50 | 1 | 0.25 | 0.75 | 0.25 | 0.25 | 0.75 | 0.50 | 0.50 | 1 | 0.50 | 0.75 | 0.25 |
| 4. Did the authors include a sample size justification? | No | No | No | No | No | No | Yes | No | No | No | No | No | No |
| 5. Were controls selected or recruited from the same population that gave rise to the cases (including timeframe)? | Yes | Yes | Unclear | Unclear | Unclear | Unclear | Yes | Yes | Yes | Yes | Yes | Yes | Yes |
| 6. Were the definitions, inclusion and exclusion criteria, used to identify or select cases and controls valid, reliable, and implemented consistently across all study participants? | Yes | Yes | No | Unclear | No | No | Yes | Yes | Yes | Yes | Yes | Yes | No |
| 7. Were the cases clearly defined and differentiated from controls? | Yes | Yes | No | No | No | No | Yes | Yes | Yes | Yes | Yes | Yes | Yes |
| 8. Were handling of specimens and pre-analytical procedures reported in sufficient detail and similar for the whole sample? | 1 | 1 | 0 | 0.50 | 1 | 0.50 | 1 | 0.50 | 0.50 | 1 | 1 | 0.50 | 0 |
| 9. Is the time period between the reference standard and the index test short enough to reasonably guarantee that the target condition did not change between the two tests? | Yes | Yes | Unclear | Yes | Yes | Unclear | Yes | Yes | Yes | Yes | Yes | Yes | Yes |
| 10. Is the reference standard likely to correctly classify the target condition? | Yes | Yes | Unclear | No | Unclear | No | Yes | Yes | No | Yes | Yes | No | Yes |
| 11. Did the whole sample or a random selection of the sample receive verification using a reference standard of diagnosis? | Yes | Yes | Yes | Unclear | Yes | No | Yes | Yes | Yes | Yes | Yes | Unclear | Yes |
| 12. Were key potential confounding variables measured and adjusted statistically in the analyses? If matching was used, did the investigators account for matching during study analysis? | 1 | 1 | 0.50 | 1 | 0.50 | 0 | 1 | 1 | 1 | 1 | 1 | 1 | 1 |
| 13. Were uninterpretable/intermediate test results reported? | No | No | No | No | No | No | Unclear | Unclear | Unclear | No | No | No | No |
| **Total score** | 10.5 | 11 | 2.75 | 4.25 | 5.75 | 1.75 | 11.75 | 10 | 9 | 11 | 10.50 | 8.25 | 8.25 |
| **Quality evaluation** | Moderate | High | Very low | Low | Low | Very low | High | Moderate | Moderate | High | High | Moderate | Moderate |
